# Prevention measures and socio-economic development result in a decrease in malaria in Hainan, China

**DOI:** 10.1186/1475-2875-13-362

**Published:** 2014-09-15

**Authors:** Shan-Qing Wang, Yu-Chun Li, Zhi-Ming Zhang, Guang-Ze Wang, Xi-Min Hu, Whitney A Qualls, Rui-De Xue

**Affiliations:** Hainan Provincial Centre for Disease Control and Prevention, Haikou, 570203 China; Haikou Centre for Disease Control and Prevention, Haikou, 571100 China; Department of Public Health Sciences, University of Miami Miller School of Medicine, Miami, FL USA; Anastasia Mosquito Control District, St Augustine, FL USA

**Keywords:** Malaria, Integrated vector management, Malaria preventative medication, Grey correlation

## Abstract

**Background:**

Historically, the incidence of malaria in the Hainan Province, China has been high. However, since 2001 the malaria incidence in Hainan has decreased due to large-scale, public educational, promotional campaigns and the adoption of preventative measures against malaria following the fast growth of socio-economic development. The present study analysed the correlation between prevention measures and social economic development on the incidence of malaria in Hainan from 2001 to 2013.

**Methods:**

The data of malaria preventative measures and socio-economic development were collected from various cities and counties in Hainan Province from 2001 to 2013 and analysed by the grey correlation analysis system.

**Results:**

Seasonal preventive medication and local fiscal revenue increases are significantly related to the reduction of malaria incidence from 2001 to 2013 (R_1_ = 0.751677; R_5_ = 0.764795).

**Conclusion:**

Malaria prevention and control measures and local economic development in Hainan decreased malaria incidence from 2001 to 2013.

## Background

Hainan Island is located in the southern part of China, with northern latitude 18°10′ ~ 20°10′, and eastern longitude 108°37′ ~ 111°03′. It has a population of 8.54 million, among which 83% are Han nationality, where 17% are minority. Hainan Island covers an area of 34 thousand square kilometer. Mountains and hills covering 38.7% of the area, mainly located at south central Island, are the main terrain of Hainan Island. The Island has an average temperature of 23.4 - 26.5°C, with annual precipitation of 1437.0 – 3022.7 mm and annual daylight duration of 1573.5 – 2443.4 hours. Weather and environment are suitable for breeding of the malarial vectors, like *Anopheles dirus* and *Anopheles minimus*.

Malaria has been reduced dramatically in China as a result of unprecedented governmental and international organizational efforts. Thirty million malaria cases were reported in 1949 in the People’s Republic of China, which has since been dramatically decreased to 1,314 cases in 2011 [1, 2]. In addition, during the same time, the epidemiological counts were decreased proportionally from 1,829 to 353 [3]. Hainan Province, located in the south of China, was one of the most important endemic malaria areas, with a high transmission of *Plasmodium falciparum* and *Plasmodium vivax*. Hainan implemented unprecedented measures for controlling malaria, such as mass drug administration (MDA) [4], long-lasting insecticide-treated mosquito nets (LLINs) [5], artemisinin-based combination therapy (ACT) [6], radical treatment [7], indoor residual spraying (IRS) [8], and chemoprophylaxis [9]. These methods have proven effective for controlling and preventing malaria transmission and there is no local- acquired case of *P. falciparum* reported in Hainan since 2009 [3].

In China, comprehensive prevention measures, including controlling malaria vectors and treating patients, were strongly encouraged and applied in the field with successful progress [10]. However, it is unclear which of the comprehensive measures implemented for controlling malaria were more effective. Statistical models, such as a logistic relation model, a negative binomial model, and a join point regress model have been applied to analyse the relationship between the incidence of malaria and climate, control measures and economic factors [11–13]. Grey relational analysis (GRA), is a useful tool for the selection of optimized factors from multiple performance characteristics, and has been applied in the field of engineering [14, 15]. GRA models are derived from the grey system theory and used as a method for analysing relationship between outcomes and factors. GRA has gradually been applied to clinical evaluation, socio-economic and natural factors on the influence of malaria epidemics and experimental studies [16, 17].

In this paper, the GRA method was used to make a comprehensive evaluation of the relationship between the incidence of malaria, human interventions in relation to malaria prevention and prevention measures following socio-economic development in Hainan, China.

## Methods

### Data resource and collection

Data were compiled from reports by the Ministry of Health and Malarial Control and Research in Hainan Province (2000–2013) on malaria incidence, the administration of primaquine, preventative measures in endemic seasons, the size of residual spraying areas, and the number of LLINs distributed [18]; collected from the Hainan Yearbook [19] were: socio- economic development of Hainan Province’s gross domestic product (GDP), GDP per capita of Hainan Province, the provincial agricultural population, the number of rural laborers, the local finance income, per capita net income of rural households, rural residents, and rural health care spending per capita housing area.

### Analysis method

The grey correlation analysis software GM (grey system theory and application of the third edition) was used [20].

To facilitate the analysis, the mean dimensionless processing model was used to sequence the comparison between the different dimensions and orders of magnitude. The formula below was applied:


where *K* is the correlation coefficient representing the moment curve and the relative difference between the reference curve. Among the curves, the coefficient of 0.5 is used to distinguish between 0 and 1 of the general selection [21].

GRA uses the incidence of a disease (1/10,000) for the associated factors (X0/Y0); X1 represents the number of preventive medicine strategies; *X*2 represents preventive medication dose rate (%); X3 represents the resting phase effect on a radical cure medication number; X4 represents the resting phase effect on a radical cure medicine suit ratio (%); X5 represents the retention area (sq m) of the insecticide spray path (IRS); X6 represents LLINs. The social development factors, in order: Y1 represents Hainan Province (100 million yuan); Y2 represents GDP per capita; Y3 represents the provincial agricultural population (10,000); Y4 represents rural laborers (10,000); Y5 represents the local fiscal revenue (10,000 yuan); Y6 represents the per capita net income of rural households; Y7 represents the rural residents’ health care expenditure; and, Y8 represents the rural per capita housing area (sq m). Data from 2001–2013 were entered into a computer software operating system item by item and the indices of correlation between the correlation factors and associated factors were conducted.

## Results

### Control measures and the incidence

**Figure 1:**
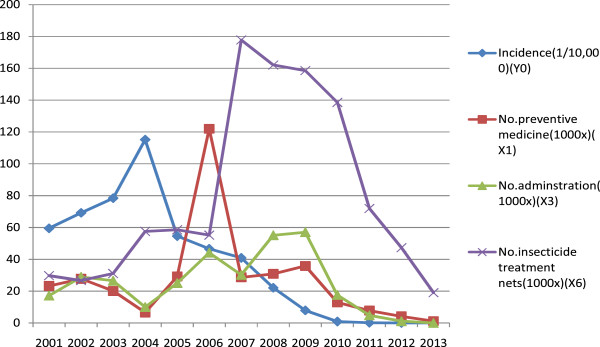
**Relationship of malaria incidence and malaria prevention and control measures in Hainan Province, China from 2001 to 2013.**

The result showed that the control measures used were significantly related to the malaria incidence in Hainan Province, China (r1 = 0.751677, r2 = 0.60305, r3 = 0.628916, r4 = 0.563998, r5 = 0.615526, r6 = 0.661795) (Table 1). The comprehensive preventative measures are ranked according to the significant impacts on malaria incidence reduction: preventative medicine (X1), radical medication (X3), LLINs (X6), IRS area (X5), prevention medicine dose rate (*X*2), and radical medicine suit ratio (X4) (Figure 1). During the ten-year period, a popular seasonal preventive medication provided to the people of Hainan had the most significant relationship with the reduction of malaria incidence.

**Table 1:** **The correlation analysis of malaria incidence, malaria prevention and control measures**

Years	Incidence	Preventive medicine (PM)		Radical treatment (RT)		Insecticides ^#^	
	(1/10,000) (X0)	No. of PM (X1)	% of PM ( ***X***2)	No. of RT (X3)	% of finish RT (X4)	Area of IRS(m ^2^) (X5)	No. of ITNs (X6)
2001	5.95	23,202	95.78	17,162	96.6	642,031	29,717
2002	6.92	27,725	96.5	28,903	93.01	0	26,669
2003	7.84	20,093	95.16	26,538	97.42	988,165	31,098
2004	11.52	6,504	96.21	10,026	99.68	869,996	57,551
2005	5.46	29,239	96.69	25,109	98.65	506,935	58,624
2006	4.66	122,024	93.04	44,203	98.88	1,049,305	55,188
2007	4.09	28,646	91.3	30,263	99.05	248,425	177,742
2008	2.21	30,818	94.16	55,091	98.79	1,257	162,064
2009	0.79	35,811	96.2	57,048	98.74	282,908	158,541
2010	0.09	12,850	94.15	17,571	97.89	470,701	138,548
2011	0.01	7,726	98.91	4,791	97.58	262,295	71,941
2012	0	415	96.26	1,143	93.16	730,452	47,356
2013	0	1,132	99.76	22	100	117,954	19,083
Correlation coefficient		0.7917	0.5527	0.7000	0.5389	0.6032	0.6592

^#^2.5% Cyhalothrin.

### Socio-economic development and malaria incidence

**Figure 2:**
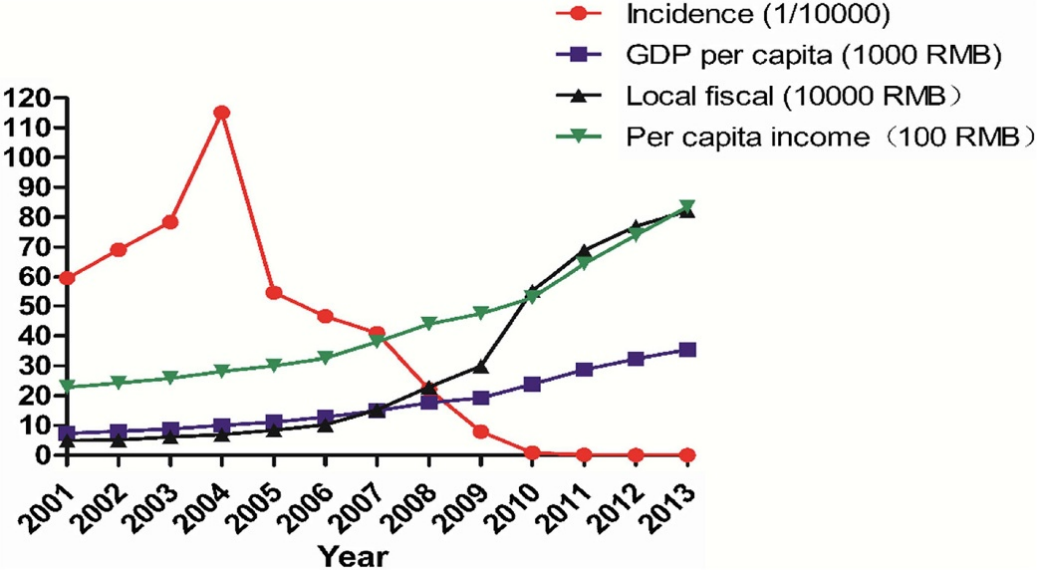
**Relationship between socio-economic development and incidence of malaria in Hainan Province, China, 2001–2013.**

The results showed that socio-economic development in Hainan Province was significantly related to malaria incidence (r1 = 0.676872, r2 = 0.666447, r3 = 0.64267, r4 = 0.598968, r5 = 0.764795, r6 = 0.645387, r7 = 0.586146, r8 = 0.635062) (Table 2).Socio-economic development has associations with the reduction of malaria incidence: local fiscal revenue (Y5) > Hainan Province revenue (Y1) > GDP per capita (Y2) > per capita net income of rural households (Y6) > rural per capita housing area (Y8) > rural resident health care expenditure (Y7) > provincial agricultural population (Y3) > rural laborers (Y4) (Figure 2).

**Table 2:** **The correlation analysis of malaria incidence with socio-economic development in Hainan Province**

Years	(Y0)	(Y1)	(Y2)	(Y3)	(Y4)	(Y5)	(Y6)	(Y7)	(Y8)
2001	5.95	579.17	7,315	567.19	229.53	495,924	2,285	39.1	19.61
2002	6.92	642.73	8,041	570.43	233.53	518,324	2,423	62.23	19.16
2003	7.84	713.96	8,849	574.9	240.27	615,971	2,588	96.05	19.51
2004	11.52	819.66	10,067	501.15	250.04	692,965	2,818	86.57	19.92
2005	5.46	918.75	11,165	505.3	256.01	848,930	3,004	93.00	21.82
2006	4.66	1,065.67	12,810	512.07	259.87	1,023,508	3,256	110.92	22.05
2007	4.09	1,254.17	14,923	521.39	269.28	1,524,579	3,791	95.55	22.64
2008	2.21	1,503.06	17,691	529.76	274.6	2,297,559	4,390	123.82	22.84
2009	0.79	1,654.21	19,254	539.31	281.59	2,996,659	4,744	129.26	24.00
2010	0.09	2,064.5	23,831	552.63	284.58	5,516,154	5,275	138.35	24.74
2011	0.01	2,515.29	28,797	434.3	225.18	6,898,400	6,446	175.52	25.35
2012	0	2,855.26	32,374	457.45	237.19	7,708,700	7,408	201.72	26.12
2013	0	3,146.46	35,317	423.11	219.38	8,211,000	8,343	227.18	26.59
Correlation coefficient	0.6551	0.6479	0.5879	0.5490	0.7073	0.6420	0.6079	0.6405	

Note: Y0-Incidence (1/10,000); Y1-GDP (100 million yuan); Y2- Real GDP per capita (yuan); Y3- Agricultural population (10,000); Y4- Rural workers (10,000); Y5- Local fiscal revenue (10,000 yuan); Y6- Per capita net income of rural households (yuan); Y7- Rural residents’ healthcare expenditure (yuan); Y8- Rural per capita housing area (sq m).

## Discussion

In 2010, the Chinese government decided to embark upon the national malaria elimination program (NMEP), with the goal of eliminating malaria by 2015 in the majority of China with the exception of the border region of Yunnan Province. In addition, the goal of elimination in the People’s Republic of China was set for 2020. Furthermore, the Action Plan of China Malaria Elimination (2010–2020) (APCME) was issued by the Chinese government [10]. According to APCME standards, there were ten counties of high malaria incidence in Hainan Province.

The major factors used to reduce malaria incidence were the promotion and administration of preventative medicines, primarily primaquine, and the distribution of LLINs before the start of malaria transmission in endemic regions. The small correlation between the ratio of completed courses of radical medical treatment and the incidence of malaria in this study may suggest that it is also necessary to administer anti-malarial drugs. Recent progress in malaria control has renewed enthusiasm and interest in MDA as a potential strategy for elimination and eradication [22–24]. MDA has also been considered as a strategy to contain the recent emergence of artemisinin resistance in the Cambodia-Thai and Thai-Myanmar borders and Jiangsu Province [10, 24, 25].

The combination of anti-malarial drugs and LLINs have been followed by reports of a decline in transmission of malaria in South Africa, Thailand, Rwanda, Ethiopia, and Zanzibar [26–29]. In China, deltamethrin-treated LLINs have reduced the density of indoor *Anopheles minimus*, a main vector in high malaria areas on Hainan Island, and reduced indoor mosquito-parasite transmission, but have not affected malaria transmission outdoors [30, 31].

Pesticide residual spraying of an area was found to have little association with the decrease in the incidence of malaria. This relationship may be caused by the broad impact of preventative and curative measures and the lack of integrated vector management strategies available in Hainan [30]. In Kenya the effect of both IRS and *Bacillus*-based larvicides reduced malaria transmission and the number of clinical malaria cases in 2010 and 2011 [32]. The feasibility of IRS malaria control in Hainan Province needs to be studied further due to the influences of indoor and outdoor resting behaviors of the major vector mosquito, *An. minimus*.

Socio-economic development has a close relationship with people’s physical health; it influences malaria incidence within the populations. According to the calculation, the results showed that the local fiscal revenue is significantly related to the malaria incidence, followed by GDP in Hainan. Socio-economic development has increased local fiscal revenue and improved farmers’ living conditions. This has directly reduced breeding sites for vector mosquitoes and drastically reduced the incidence of malaria. However, since there was little correlation between expenditure of rural residents and malaria incidence, this may indicate that farmers have not paid enough for medical care or healthcare expenses related to malaria treatment.

In addition to control and prevention measures, improving social and economic conditions, especially for ethnic minorities and in remote rural areas, is essential for malaria elimination in Hainan. The establishment of a network of medical treatment which provides a combination of malaria prevention and control along with basic public health services is necessary; promotion of health education and healthcare awareness are priorities for malaria control for minority groups in remote areas of Hainan Province, China [33, 34].

## Conclusion

The promotion of preventative measures through the administration of anti-malarial drugs, LLINs and radical treatment medication have benefited malaria control in Hainan Province, China. Socio-economic development, such as local and provincial economic growth and improved health conditions, have significantly reduced malaria incidence in Hainan Province, China from 2001 to 2013.

## Abbreviations

ACT: Artemisinin-based combination therapy
GDP: Gross domestic product
GRA: Grey relational analysis
IRS: Indoor residual spraying
LLINS: Long-lasting, insecticide-treated mosquito nets
MDA: Mass drug administration
NMEP: National malaria elimination program
APCME: Action plan of China malaria elimination.
